# Simulation-based team training at the sharp end: A qualitative study of simulation-based team training design, implementation, and evaluation in healthcare

**DOI:** 10.4103/0974-2700.70754

**Published:** 2010

**Authors:** Sallie J Weaver, Eduardo Salas, Rebecca Lyons, Elizabeth H Lazzara, Michael A Rosen, Deborah DiazGranados, Julia G Grim, Jeffery S Augenstein, David J Birnbach, Heidi King

**Affiliations:** Department of Psychology and Institute for Simulation and Training, University of Central Florida, Orlando Florida, USA; 1 William Lehman Injury Research Center, Miami, Florida, USA; 2 University of Miami-Jackson Memorial Hospital Center for Patient Safety, University of Miami Miller School of Medicine, Miami, Florida, USA; 3 Office of the Assistant Secretary of Defense (Health Affairs) TRICARE Management Activity, Falls Church, Virginia, USA

**Keywords:** Simulation-based training, teams, teamwork

## Abstract

This article provides a qualitative review of the published literature dealing with the design, implementation, and evaluation of simulation-based team training (SBTT) in healthcare with the purpose of providing synthesis of the present state of the science to guide practice and future research. A systematic literature review was conducted and produced 27 articles meeting the inclusion criteria. These articles were coded using a low-inference content analysis coding scheme designed to extract important information about the training program. Results are summarized in 10 themes describing important considerations for what occurs before, during, and after a training event. Both across disciplines and within Emergency Medicine (EM), SBTT has been shown to be an effective method for increasing teamwork skills. However, the literature to date has underspecified some of the fundamental features of the training programs, impeding the dissemination of lessons learned. Implications of this study are discussed for team training in EM.

## INTRODUCTION

Healthcare is entering a new era characterized by an unfreezing of old processes, attitudes, and approaches. Instantiation of progressive, participatory care approaches requires examination of existing evidence to ascertain what is (and what is not) advancing the provision of quality care. This new era is underlain by several recent shifts in basic assumptions regarding the route to effective care in a healthy work environment, especially in critical areas like Emergency Medicine (EM). We focus specifically on two of these basic assumptions. First, that teamwork is a critical component of a safe healthcare system, in general,[1–3] as well as in EM specifically due to its dynamic, high risk nature[4–7] and second, that SBTT a critical component of implementing effective and teamwork training that is highly transferable to the daily clinical environment.[8–10]

Efforts to optimize teamwork have most commonly focused on training teams on the knowledge, skills, and attitudes (KSAs) underlying effective team performance.[11,12] Recognizing the opportunities offered by SBT, team training content is being integrated with SBT methods to provide teams with vital opportunities to develop, practice, and refine core teamwork competencies without the threat of patient harm. However, the knowledge of such innovative work is often disseminated through non-peer-reviewed sources, not reported in adequate detail, or not presented publically at all. To continue the advancement of simulation-based team training (SBTT) in healthcare, we must be able to identify what work is being done, what is effective, and what remains to be tested.

Therefore, in an attempt to delve deeper into the existing evidence, this article presents a qualitative analysis of the existing peer-reviewed literature on healthcare SBTT in order to synthesize critical themes from the most rigorously reported studies to date. Specifically, we review how simulation has been integrated into the design, implementation, and evaluation of SBTT. Published SBTT studies are relatively few in number; thus, our review spans across clinical domains and results are presented in terms of 10 primary themes. In detailing each theme, we draw upon the broader simulation literature to contribute to interpretation and practical recommendations specifically addressing issues in EM. Ultimately, we hope to contribute a view of the state of the science and practice of improving patient safety with SBTT in healthcare.

## WHAT IS SIMULATION-BASED TRAINING?

SBT is an instructional technique designed to accelerate expertise by allowing for skill development, practice, and feedback in settings replicating real world clinical environments.[13] Simulation fosters effective learning through active learner engagement, repetitive practice, the ability to vary difficulty and clinical complexity, as well as diagnostic performance measurement and intra-experience feedback.[14,15] Additionally, even simulations relatively low in physical fidelity have demonstrated validity as an approximation of the clinical practice environment. The critical factor is that the simulation scenario induces transfer-appropriate processing; that is, those cognitive processes required for performing a task under normal operating conditions.[16,17] Regardless of fidelity, simulation provides a safe, yet realistic mechanism for developing and fine tuning skills without serious consequential risk. For these reasons, SBT is a popular method among both trainees and trainers. From the trainee perspective, SBT is generally perceived as a useful and well-liked training experience.[18]

Though trainee reactions are not necessarily indicative of training effectiveness, they can greatly influence trainee engagement and effort during training, and thus contribute to learning. In turn, opportunities for development such as SBT have also been linked with important patient outcomes and organizational level outcomes such as employee retention.[19]

SBT is effective and well received in part because it incorporates multiple learning modalities (i.e., information, demonstration, and practice). Traditional didactic training is limited in its ability to generate transfer appropriate processing—trainees have no opportunity to actively engage, practice, and refine the cognitive, affective, and behavioral strategies necessary for utilizing newly trained skills in daily practice. While SBT generally includes an informational (i.e., didactic) component, these programs also incorporate demonstration and multiple opportunities for cognitive and kinesthetic practice of targeted competencies. Thus, SBT can accommodate individual differences in preferred learning style[20] and can be adapted to suit the needs of a specific context or training population.

Expanding upon SBT which emphasizes technical clinical skills, SBTT seeks to advance those non-technical teamwork skills underlying effective team communication, cooperation, and coordination such as closed-loop communication, situational awareness, back-up behaviors, as well as necessary supportive structures such as shared mental models.[21,22] Recognizing the essential nature of quality teamwork, healthcare has embraced team training, yet SBTT training methodologies remain underutilized and their effectiveness relatively underreported.[23,24]

To help address this gap, the remainder of this article is dedicated to detailing key themes emerging before, during, and after SBTT in the available peer reviewed healthcare literature. A brief discussion of the review process is presented prior to discussion of these themes.

## MATERIALS AND METHODS

The articles reviewed in the current study represent a subset of a larger, more comprehensive dataset designed to capture all forms of team training utilized in healthcare, not only simulation-based studies. We refer readers to the original source for explicit details of the literature search.[23] Inclusion criteria for the current review required that studies (1) were reported in the peer-reviewed literature, (2) implemented a team-training program that was primarily simulation-based, (3) reported evaluation data, and (4) reported adequate details regarding the description of the SBTT and related evaluation data. Studies which reported evaluation data, but did not provide a description of actual training content, structure, implementation, or a citation where this information was previously published were not included. In cases where another study was cited for additional information regarding training design, this information was coded from the secondary source.

Twenty-seven studies (*N* = 27) meeting these criteria were coded using a detailed, low inference coding framework established to extract key elements regarding training design, participants, implementation, evaluation, and any author reported guidelines or lessons learned regarding SBTT (See appendix). Coded content was analyzed using content analysis, a method for analyzing text data affording an opportunity to generate categories, themes, and patterns.[25]

## RESULTS

Results are summarized in Tables 1–3; however, a detailed presentation of extracted findings appears below.

**Table 1:** Summary of qualitative review results before training

Training needs analysis reported	Yes	No	–	–	–	–
*n*	4	23	–	–	–	–
% of 27articles	14.8	85.2	–	–	–	–
Baseline individual proficiency assessed	Yes	No	–	–	–	–
*n*	6	21	–	–	–	–
% of 27articles	22.2	77.8	–	–	–	–
Training objectives specified	Yes	No	–	–	–	–
*n*	10	17	–	–	–	–
% of 27 articles	37.0	63.0	–	–	–	–
Training focus: teamwork vs. taskwork	Teamwork focus	Combination	–	–	–	–
*n*	19	8	–	–	–	–
% of 27 articles	70.4	29.6	–	–	–	–
Teamwork focused training content	Communication	Role clarity	Situational awareness	Leadership	–	–
*n*	21	9	13	11	–	–
% of 27 articles	77.8	33.3	48.1	40.7	–	–
Type of providers targeted for training	Physicians and nurses	Nurses only	Students/ residents	Multiple disciplines	Other	Not specified
*n*	6	4	5	9	2	1
% of 27 articles	22.2	14.8	18.5	33.3	7.4	3.7
Specialty targeted for training	Pediatrics	Anesthesia	Emergency	Other	Not specified	–
*n*	2	5	6	11	3	–
% of 27 articles	7.4	18.5	22.2	40.7	11.1	–

**Table 2:** Summary of qualitative review results during training

Type of team training	CRM	Team-building	Cross training	Goal setting	Combination	Not specified
*n*	18	1	1	2	1	4
% of 27 articles	66.7	3.7	3.7	7.4	3.7	14.8
Instructional methodology	Simulation only	Mixed instructional methods	Not specified	–	–	–
*n*	2	25	0	–	–	–
% of 27 articles	7.4	92.6	0.0	–	–	–
Simulation fidelity	High fidelity simulation	Low fidelity simulation	–	–	–	–
*n*	16	11	–	–	–	–
% of 27 articles	59.3	40.7	–	–	–	–
Who facilitated training?	Internal medical personnel	Outside trainer	Authors	Mix	Not specified	–
*n*	7	4	1	1	14	–
% of 27 articles	25.9	14.8	3.7	3.7	51.9	–
Team size	Small (2 members)	Medium (3-5)	Large (5 or more)	Not specified	–	–
*n*	4	10	3	10	–	–
% of 27 articles	14.8	37.0	11.1	37.0	–	–
Team familiarity	Intact	Ad-hoc	Did not specify	–	–	–
*n*	6	5	16	–	–	–
% of 27 articles	22.2	18.5	59.3	–	–	–
Feedback	Feedback given	Not specified	–	–	–	–
*n*	20	7	–	–	–	–
% of 27 articles	74.1	25.9	–	–	–	–

**Table 3:** Summary of qualitative review results after training

Training evaluation	Single level evaluation (i.e. trainee reactions only)	Multi-level evaluation	–	–	–
*n*	4	23	–	–	–
% of 27 articles	14.8	85.2	–	–	–
Evaluation criteria	Reactions	Knowledge	Behavior	Patient of organizational outcomes	–
*n*	16	9	20	5	–
% of 27 articles	59.3	33.3	74.1	18.5	–
Evaluation timeframe	Immdiately	3 months later	6 months later	Not specified	Evaluated at multple points
*n*	20	3	4	4	4
% of 27 articles	74.1	11.1	14.8	14.8	14.8

### Before training

The science of training underscores that several factors are important during training development.[14,26,27] Specifically, our review focused on training needs analysis, the competency areas targeted for training, and the types of providers targeted for training.

#### Training needs analysis

A thorough needs analysis is vital to understanding who to train, what to train, and how best to deliver training. Generally, training needs analyses are composed of three dimensions: organizational analysis (i.e., what are focal organizational (or unit) goals and opportunities for improvement?), task analysis (i.e., what tasks and underlying KSAs are needed for effective, efficient job performance?), and person analysis (i.e., what is the current level of KSAs and performance? Is there room for improvement compared to levels identified in the task analysis?). In the current review, only 15% (*n* = 4) of studies explicitly indicated that some form of needs analysis was conducted in order to feed training development. For 85% of the reviewed articles (*n* = 23), no insight was provided regarding how it was determined that team training was the “right treatment” for the targeted providers and that simulation was the most optimal training methodology. Additionally, only 22% (*n* = 6) reported that baseline levels of teamwork skills or experience were assessed for targeted trainees.

#### Competency areas targeted for training

A sound conceptual model of teamwork should underlie the development of training content. While no single overarching general model of teamwork captures all aspects of teamwork within a given clinical specialty or for a specific team, it is important that targeted teamwork competences are transportable and generalizable considering that most healthcare workers spend their daily lives working as members of multiple teams.[28] For example, reviewed studies tended to target communication (78%, *n* = 21), situational awareness (48%, *n* = 13), leadership (40%, *n* = 11), and role clarity (33%, *n* = 9). These competency areas align with critical models of teamwork both within healthcare and the broader organizational literature.[29,30] Additionally, 70% (*n* = 19) focused training solely on teamwork skills. While there is often a push to squeeze as much content as possible into training opportunities, introducing new clinical skills during team training severely limits its validity as a mechanism for developing teamwork skills.

#### Providers targeted for training

In line with the core principles of team training, almost all of the reviewed studies took a multidisciplinary approach. Twenty-two percent (*n* = 6) of reviewed articles targeted physicians and nurses only; however, over 33% (*n* = 9) focused on a broader range of providers. SBTT programs have included nurses, physicians, midwives, technicians, and orderlies.[31] While EM was the most commonly targeted specialty (22%, *n* = 6), reviewed studies targeted specialties spanning primary care to geriatrics, suggesting SBTT as a viable mechanism for optimizing teamwork across a broad range of clinical arenas.

### During training

In terms of understanding how SBTT is being carried out, our review focused on team training strategy, how training methods were integrated in content delivery (i.e., information, demonstration, and simulation), team size and familiarity, and the provision of feedback.

#### Training strategy

Training strategies are defined as overarching curriculum approaches which combine content and training methods into an overarching training intervention. One of the most well-known training strategies is Crew Resource Management (CRM), a strategy originating in the aviation community, designed to increase team reliability and reduce errors by optimizing teamwork and teaching team members to maximally utilize all available resources.[32] CRM targets a wide range of behaviors from communication, assertiveness, leadership, and decision making to situational awareness and adaptability.[33,34] This strategy also stimulates high reliability by fostering realistic awareness of personal limitations and abilities.

In the current review, CRM was the most commonly utilized training strategy, that is, 67% (*n* = 18) of reviewed articles explicitly noted utilizing a CRM derived training strategy. For example, Gaba and colleagues[35] adapted traditional aviation CRM principles to form the Anesthesia Crisis Resource Management (ACRM) training strategy. ACRM employs high fidelity simulation to train anesthesiologists to maintain situational awareness, prevent and manage fixation errors, foster effective communication, provide work load management techniques, and offer strategies for optimal management of available resources. Furthermore, strategies such as ACRM focus on the recognition and management of errors, providing scenarios in which trainees have the opportunity to practice recovering from errors in a non-consequential environment.[36]

#### Integration of training delivery methods

Ninety-three percent of the reviewed articles (*n* = 25) integrated multiple modes of instruction, combining simulation with other instructional methodologies such as didactic instruction,[37] video models of targeted skills,[38] and live demonstration.[39] While the majority of reviewed studies relied on mixed methods of instruction, 7% (*n* = 2) were conducted exclusively through the use of simulation. Furthermore, our review underscored that SBTT does not equal expensive simulation laboratories with high physical fidelity. In fact, only 59% (*n* = 16) of reviewed studies conducted training in such environments. The remainder leveraged low fidelity simulations such as role playing.

In terms of training facilitation, 26% (*n* = 7) of SBTT programs were led by in-house medical personnel and 15% (*n* = 4) were led by an external trainer. However, 52% (*n* = 14) of studies did not specify who facilitated the SBTT program.

#### Team size and familiarity

Only 17 studies explicitly discussed team size. Of these, the majority (59%, *n* = 10) conducted training using medium-sized teams consisting of three to five members. Additionally, of the 27 coded articles, 11 specified team familiarity. Of these, 55% (*n* = 6) conducted SBTT using intact teams, those teams who work together on a regular basis and enter the training environment with a pre-existing level of familiarity with one another. Nineteen percent (*n* = 5) conducted training using *ad hoc* teams comprising individuals brought together as a team for the first time during training.

#### Training duration

Training duration varied from 3 hours to several days spread across a period of multiple months. While 11% (*n* = 3) of studies did not indicate training duration, 59% (*n* = 16) specified that training lasted less than 1 day, and 30% (*n* = 8) indicated that training was spread across multiple days.

#### Feedback

Seventy-four percent (*n* = 20) of reviewed studies indicated that diagnostic feedback was provided to trainees. Of these, 100% (*n* = 20) indicated that feedback was provided face-to-face, and the majority (60%, *n* = 12) noted that feedback focused on actual behaviors observed during simulation scenarios (i.e., processes). For example, Stroller and colleagues[40] incorporated both process and outcome-based feedback in their debriefings, which allowed participants to objectively compare their individual performance score to the team’s overall performance score. While outcome-based feedback alone does not enable diagnosis of why certain outcomes occurred, outcome-based debriefings can be perceived as more objective; therefore, combining process and outcome feedback from multiple sources (e.g., self, peers, facilitator) can be useful in presenting the most complete and valid picture of performance.

In terms of the timing of feedback, 16 of the 20 studies that described how feedback was provided to trainees indicated at what point in the training process feedback was provided to trainees. Of these, 94% (*n* = 15) indicated that feedback was provided via a post-training scenario debriefing. For example, Shapiro and colleagues[41] followed each simulation scenario with a comprehensive, facilitated, and structured debriefing in which team members focused discussion on teamwork processes. Each team’s performance during the simulation was video recorded, and the tape was reviewed during each debriefing session in order to allow team members the opportunity to engage in self-assessment and reflection by identifying team success and opportunities for improvement.

### After training

Evaluation is a critical component of SBTT. Therefore, in exploring what is being reported after SBTT implementation, our review focused on determining the prevalence of multilevel evaluation, what types of outcomes were more commonly being targeted, and when evaluation data are being collected.

#### Multilevel evaluation and types of outcomes targeted

Classic frameworks for training evaluation[42] outline several levels at which training programs can be evaluated: (1) reactions (i.e., level of satisfaction, liking, perceived usefulness), (2) learning (i.e., knowledge gains and/or attitude changes), (3) behaviors (i.e., in simulated performance environments and on the job), and (4) results (i.e., the degree to which the behaviors learned training and enacted on the job produce value for the patient and/or organization). While much of the early SBTT literature has been criticized for only evaluating reactions, 85% (*n* = 23) of studies reported multilevel evaluations that assessed criteria beyond how much trainees liked or were satisfied with the program. Specifically, 74% (*n* = 20) assessed trainee behavior, 33% (*n* = 9) assessed trainee learning, and 19% (*n* = 5) assessed patient or organizational outcomes. For example, following the implementation of SBTT among operating room teams, Awad and colleagues[43] reported a significant improvement in the number of appropriate patients receiving deep venous thrombosis (DVT) prophylaxis before induction, in the administration of prophylactic antibiotics within 60 minutes of incision, in appropriate use of sequential compression devices, in identifying high risk patients prior to performing an operation, in a decrease of adverse events, and improved patient safety.

#### Timeframe for evaluation

While 15% (*n* = 4) did not specify when evaluation data were collected, 74% (*n* = 20) of reviewed studies collected their primary evaluation data immediately after training. Several studies (15%, *n* = 4) also collected longitudinal data up to 6 months post-training.

## SPECIFIC CONSIDERATIONS FOR SIMULATION-BASED TEAM TRAINING IN EMERGENCY MEDICINE

While results reported thus far represent a cross-disciplinary sample of SBTT studies conducted across a variety of clinical settings, the existing evidence does provide some explicit insight and guidance for EM. Overall, SBTT has demonstrated effectiveness within the EM clinical environment. For example, Shapiro and colleagues[40] implemented SBTT within a level 1 trauma center located in a major academic hospital. While they reported that participants (i.e., nurses, technicians, EM residents, and attending physicians) all rated the SBTT as a useful educational tool, they also found significant improvements in teamwork behavior occurring during simulated trauma events. Similarly, DeVita *et al*.[44] successfully implemented SBTT to improve medical emergency team (MET) performance for trauma resuscitations and found that mannequin survival improved 90% across three simulation sessions.

While SBTT has been demonstrated as an effective method for enhancing teamwork skills among emergency medical providers, emergency care presents several unique considerations. Few other clinical areas provide care under the time-pressure and ambiguity inherent in emergency care. Additionally, patient encounters are highly unique in that they are usually an encounter between two strangers (one of which is often unconscious or unable to communicate verbally) in a chaotic and emotionally charged environment in which information is being provided from multiple sources.[45] Furthermore, such information is often inconsistent and conflicting.

When considering SBTT to optimize teamwork skills within such an environment, it is imperative to account for elements that would impact training development, implementation, and evaluation. For example, one important factor to consider is that emergency medical team membership is highly dynamic, fluctuating nearly continually during most care episodes. Therefore, it is particularly critical to determine who will need to be trained. Simply training physicians and nurses would likely be insufficient for training such teams since additional ancillary staff e.g. (respiratory therapists, mobile imaging technicians, etc.) is of paramount importance to successfully manage patients.

In addition to determining who needs training, another important element is to establish the skill level of all of the trainees. Emergency medical teams are inherently multidisciplinary; team members bring different professional backgrounds, levels of expertise, skills sets, and experiences to bear on team interactions. In order for SBTT to be effective, scenarios must be developed to match the existing clinical skill levels to ensure that each learner is challenged appropriately.

Another important factor to consider is the types of cases providers encounter in the ER. Unlike other specialties, such as an orthopedic surgeon who would treat similar cases consistently, EM providers frequently treat a highly diversified case load. For example, one provider may treat sepsis, myocardial infarction, and major trauma, all within one shift. Hence, simulation scenarios must be designed to reflect this diversity by integrating commonly encountered scenarios (e.g., myocardial infarction) with high acuity/low frequency cases (e.g., pediatric lead poisoning). Additionally, scenarios should incorporate cases with fatal (e.g., resuscitation) and trivial outcomes (e.g., reflux esophagitis). Integrating a variety of scenarios will provide learners with ample opportunities to exhibit the desired teamwork skills.

In addition to the critical design criteria previously mentioned, it is also essential to evaluate actual team behaviors, both during and after training with measurement tools rooted in the science of learning and training. Several observational checklists designed to assess teamwork performance in clinical settings have been published to date (e.g. observational teamwork assessment of surgery [OTAS] and communication and teamwork skills assessment [CATS]). For example, Rosen and colleagues[46,47] developed a theoretically based approach for designing simulation scenarios and corresponding observational tools specifically designed for measuring team performance in simulation scenarios, the Simulation Module for Assessment of Resident Targeted Event Responses (SMARTER). The SMARTER approach outlines an event-based method for developing simulation scenarios in which critical trigger events are created based upon the KSAs underlying targeted teamwork competencies. These events are embedded within the simulation scenario as triggers designed to provide trainees with the opportunity to exhibit targeted team behaviors. These events, in turn, are used to create an event-based checklist which can be used to assess whether trainees exhibited desired observable, behavioral responses throughout the course of the simulation session. Performance criteria are determined *a priori* and are simply marked as being either present (i.e., hit) or absent (i.e., miss). Although a dichotomous rating system does not afford for rating the quality of behaviors, it can be beneficial for reducing the cognitive workload raters can experience when completing Likert-type ratings.

## DISCUSSION

Our review of the literature suggests that SBTT in healthcare is at a promising crossroads. In several respects, SBTT is being designed, implemented, and evaluated according to core principles of the science of training and adult learning. Training is targeting multidisciplinary team members across the healthcare spectrum. It is being designed to target critical teamwork competencies outlined in theoretical models of teamwork and is immersing trainees in scenarios that enable them to practice the KSAs underlying these competencies. Programs have incorporated comprehensive debriefing strategies in order to reinforce desired behaviors and correct undesirable behaviors. Finally, SBTT is being evaluated at multiple levels across a range of indicators.

However, our review also suggests several critical needs which researchers, medical educators, providers, and administrators must consider in future SBTT endeavors. For example, very few studies report information regarding how training needs were determined. Need analysis is vital for training development and effectiveness. It is the mechanism for ensuring that training is focused upon the “correct” team competencies underlying effective team performance in a given care context. If the training is targeting the wrong competencies, those with an insignificant bearing on team performance in a given clinical context, then the training will inherently be neither viable nor effective. Additionally, a significant proportion of current SBTT programs in the published literature are targeting intact teams who enter training with a pre-existing level of familiarity with one another’s skills, personality, preferences, and other personal characteristics. Studies of care processes across a wide variety of clinical settings, however, draw attention to the fact that many teams should be classified as *ad hoc* teams. Scheduling, variations in the knowledge and skills needed to effectively manage different cases, and other factors often result in dynamic team membership during a single care episode, leading many teams to be composed of at least some members who are unfamiliar with each other.[48,49] If teams are working in an *ad hoc* manner, but being trained as intact teams, transfer of trained skills to the daily work environment will be very limited. For many care contexts, SBTT must be designed to target generalizable team competencies which can be transported from team to team, and it must be implemented in a manner which allows team members to practice these competencies in an *ad hoc* environment. Additionally, our review indicated that most SBTT programs were training teams of three to five members. However, several studies have reported much larger average team sizes throughout several clinical areas. For example, trauma teams have been documented as ranging in size from three to nine or more,[50] and Cassera and colleagues[51] recently reported that the laparoscopic surgical procedures involved an average of eight team members (range 4–15).

Most importantly, our review underscored the need for more thorough and standardized reporting of SBTT programs and efforts to evaluate these programs. Rigorous, detailed reporting is required of clinical effectiveness research—why should we not apply similar standards to research on quality improvement strategies such as SBTT considering their significant role in quality care? To this end, authors should reference the guidelines for designing and reporting quality improvement (QI) initiatives published by Davidoff and colleagues[52] as they offer explicit guidance for detailed, comprehensive QI reporting and publication.

## CONCLUSIONS

Overall, bringing together highly skilled groups of clinicians to care for a patient will ensure that there is the most expertise possible in the room. However, if this group does not also know how to coordinate this or does not feel their skills and actions or feel motivated to work as a team, then the benefits of this benefits of this pooled expertise expertise benefits of this pooled expertise will go realized. SBTT offers a viable mechanism for ensuring that healthcare comprises clinicians who are not only technical experts but also expert team members. In our review we have striven to offer insight into what is currently happening in SBTT and present recommendations for continuing to advance this evidence base to ensure that SBTT continues to grow as a viable and an efficient method for developing the critical teamwork expertise clinicians and administrators will need as the US healthcare system enters a new era.

## Appendix

**Table:** List of coded articles

Awad SS, Fagan SP, Bellows C, Albo D, Green-Rashad B, De la Garza M, *et al*. Bridging the communication gap in the operating room with medical team training. Am J Surg 2005;190:770-4. Berkenstadt H, Haviv Y, Tuval A, Shemesh Y, Megrill A, Perry A, *et al*. Improving handoff communications in critical care: utilizing simulation-based training toward process improvement in managing patient risk. Chest 2008;134:158-62. Blum RH, Raemer DB, Carroll JS, Dufresne RL, Cooper JB. A method for measuring the effectiveness of simulation-based team training for improving communication skills. Anesth Analg 2005;100:1375-80. *Blum RH, Raemer DB, Carroll JS, Sunder N, Feinstein DM, Cooper JB. Crisis resource management training for an anesthesia faculty: A new approach to continuing education. Med Educ 2004;38:45-55. Cashman SB, Reidy P, Cody K, Lemay C. Developing and measuring progress toward collaborative, integrated, interdisciplinary health care teams. J Interprof Care 2004;18:183-96. Cole KD, Campbell LJ. Interdisciplinary team training for occupational therapists. Phys Occup Ther Geriatr 1986;4:69-74. Cooley E. Training an interdisciplinary team in communication and decision-making skills. Small Group Res 1994;25:5-25. DeVita MA, Schaefer J, Lutz J, Wang H, Dongilli T. Improving medical emergency team (MET) performance using a novel curriculum and a computerized human patient simulator. Qual Saf Health Care 2005;14:326-31. France DJ, Stiles R, Gaffney EA, Seddon MR, Grogan EL, Nixon WR Jr, *et al*. Crew resource management training--Clinicians’ reactions and attitudes. AORN J 2005;82:214-28. Gaba DM, Howard SK, Flanagan B. Assessment of clinical performance during simulated crisis using both technical and behavioral ratings. Anesthesiology 1998;89:3-18. *Holzman RS, Cooper JB, Gaba DM, Philip JH, Small SD, Feinstein D. Anesthesia crisis resource management: Real-life simulation training in operating room crises. J Clin Anesth 1995;7:675-87. Gibson CB. Me and us: Differential relationships among goal-setting training, efficacy and effectiveness at the individual and team level. J Organ Behav 2001;22:789-808. Grogan EL, Stiles RA, France DJ, Speroff T, Morris JA Jr, Nixon B, *et al*. The impact of aviation-based teamwork training on the attitudes of health-care professionals. J Am Coll Surg 2004;199:843-8. Haller G, Garnerin P, Morales MA, Pfister R, Berner M, Irion O, *et al*. Effect of crew resource management training in a multidisciplinary obstetrical setting. Int J Qual Health Care 2008;20:254-63. Gardi T, Christensen UC, Jacobsen J, Jensen PF, Ording H. How do anesthesiologists treat malignant hyperthermia in a full-scale anaesthesia simulator? Acta Anaesthesiol Scand 2001;45:1032-5. Jacobsen J, Lindekaer AL, Ostergaard HT, Nielsen K, Ostergaard D, Laub M, *et al*. Management of anaphylactic shock evaluated using a full-scale anaesthesia simulator. Acta Anaesthesiol Scand 2001;45:315-9. Marshall DA, Manus DA. A team training program using human factors to enhance patient safety. AORN J 2007;86:994-1011. Moorthy K, Munz Y, Forrest D, Pandey V, Undre S, Vincent C, *et al*. Surgical crisis management skills training and assessment: A simulation-based approach to enhancing operating room performance. Ann Surg 2006;244:139-47. Murray WB, Jankouskas T, Chasko-Bush M, Liu W, Sinz L. Crisis resource management: Anesthesia non-technical skills (ANTS) in pediatric nurses and residents. Anesthesiology 2006;105:A13325. O’Donnell J, Fletcher J, Dixon B, Palmer L. Planning and implementing an anaesthesia crisis resource management training course for student nurse anesthetists. CRNA 1988;9:50-8. Østergaard HT, Østergaard D, Lippert A. Implementation of team training in medical education in Denmark. Qual Saf Health Care 2004;13:i91-5. Reznek M, Smith-Coggins R, Howard S, Kiran K, Harter P, Sowb Y, *et al*. Emergency medicine crisis resource management (EMCRM): A pilot study of a simulation-based crisis management course for emergency medicine. Acad Emerg Med 2003;10:386-9. Robertson B, Schumacher L, Gosman G, Kanfer R, Kelley M, DeVita M. Simulation-based crisis team training for multidisciplinary obstetric providers. Simul Healthc 2009;4:77-83. Sehgal NL, Fox M, Vidyarthi AR, Sharpe BA, Gearhart S, Bookwalter T, *et al*. A multidisciplinary Teamwork Training Program: The Triad for Optimal Patient Safety (TOPS) Experience. J Gen Intern Med 2008;23:2053-7. Shapiro MJ, Morey JC, Small SD, Langford V, Kaylor CJ, Jagminas L, *et al*. Simulation based teamwork training for emergency department staff: Does it improve clinical team performance when added to an existing didactic teamwork curriculum? Qual Saf Health Care 2004;13:417-21. Sica GT, Barron DM, Blum R, Frenna TH, Raemer DB. Computerized realistic simulation: A teaching module for crisis management in radiology. Am J Roentgenol 1999;172:301-4. Stroller JK, Rose M, Lee R, Dolgan C, Hoogerf BJ. Teambuilding and leadership training in an internal medicine residency training program. J Gen Intern Med 2004;19:692-705. Wallin CJ, Meurling L, Hedman L, Hedegard J, Fellander-Tsai L. Target-focused medical emergency team training using a human patient simulator: Effects on behaviour and attitude. Med Educ 2007;41:173-80. Youngblood P, Harter PM, Srivastava S, Moffett S, Heinrichs WL, Dev P. Design, development, and evaluation of an online virtual emergency department for training trauma teams. Simul Health 2008;3:154-60.

*NOTE*: *DENOTES AN ARTICLE COMBINED WITH THE PRECEDING ARTICLE FOR CODING PURPOSES BECAUSE THE ARTICLES DESCRIBED THE SAME TRAINING PROGRAM
